# Mixed Reality in the Operating Room: A Systematic Review

**DOI:** 10.1007/s10916-024-02095-7

**Published:** 2024-08-15

**Authors:** Renato Magalhães, Ana Oliveira, David Terroso, Adélio Vilaça, Rita Veloso, António Marques, Javier Pereira, Luís Coelho

**Affiliations:** 1 LabRP-CIR, ESS, Polytechnic of Porto, Rua Dr. António Bernardino de Almeida, 4200-072 Porto, Portugal; 2 CAC ICBAS-CHP – Centro Académico Clínico Instituto de Ciências Biomédicas Abel Salazar – Centro Hospitalar Universitário de Santo António, Porto, Portugal; 3 ISEP, Polytechnic of Porto, Rua Dr. António Bernardino de Almeida, 4249-015 Porto, Portugal; 4 Executive Board Member, Centro Hospitalar Universitário de Santo António, Porto, Portugal; 5 Universidade da Coruña, CITIC Research Center, Talionis Research Group. A Coruña, La Coruña, Spain; 6 INESC TEC , Institute for Systems and Computer Engineering Technology and Science, Porto, Portugal

**Keywords:** Mixed reality, Medical remote assistance, Extended reality, augmented reality, Operating rooms, Surgery

## Abstract

Mixed Reality is a technology that has gained attention due to its unique capabilities for accessing and visualizing information. When integrated with voice control mechanisms, gestures and even iris movement, it becomes a valuable tool for medicine. These features are particularly appealing for the operating room and surgical learning, where access to information and freedom of hand operation are fundamental. This study examines the most significant research on mixed reality in the operating room over the past five years, to identify the trends, use cases, its applications and limitations. A systematic review was conducted following the Preferred Reporting Items for Systematic Reviews and Meta-Analysis (PRISMA) guidelines to answer the research questions established using the PICO (Population, Intervention, Comparator and Outcome) framework. Although implementation of Mixed Reality applications in the operations room presents some challenges, when used appropriately, it can yield remarkable results. It can make learning easier, flatten the learning curve for several procedures, and facilitate various aspects of the surgical processes. The articles’ conclusions highlight the potential benefits of these innovations in surgical practice while acknowledging the challenges that must be addressed. Technical complexity, equipment costs, and steep learning curves present significant obstacles to the widespread adoption of Mixed Reality and computer-assisted evaluation. The need for more flexible approaches and comprehensive studies is underscored by the specificity of procedures and limited samples sizes. The integration of imaging modalities and innovative functionalities holds promise for clinical applications. However, it is important to consider issues related to usability, bias, and statistical analyses. Mixed Reality offers significant benefits, but there are still open challenges such as ergonomic issues, limited field of view, and battery autonomy that must be addressed to ensure widespread acceptance.

## Introduction

### Context

Surgical practice and intraoperative spaces have evolved with technological advances, aiming for safer, faster, and more efficient methods in the operating room (OR) [1, 2]. The development of smartphones, tablets, and head-mounted displays has increased interest in using these devices for easily accessible information in the OR [3, 4].

Augmented Reality (AR) and Mixed Reality (MR) are emerging as powerful tools in surgery, promising to introduce a new era of enhancement. These innovative technologies have the potential to revolutionize the OR by seamlessly integrating digital information into the surgeon's field of view, transforming the way they learn, plan, and execute procedures.

For example, in the context of surgical procedures, monitors are skillfully managed by qualified operating room personnel who have both the expertise and resources necessary to prioritize the surgeon's optimal performance. However, certain intricate aspects of surgery require the deep insight that only the surgeon can provide [5–7]. While it is possible for the surgeon to ask someone to look up information, this introduces the need for additional personnel, increasing costs and infection risks, and requiring the availability of skilled staff. Using a tablet or smartphone during surgical planning is also an option, but in the OR, maintaining aseptic conditions is critical. This is where MR can offer significant advantages, it provides hands-free, autonomous access to information, allowing surgeons to make critical decisions quickly and accurately, such as selecting the optimal angle for a screw insertion. Unlike a tablet, MR devices are sterile and offer surgeons autonomy and faster information access, making them a superior tool in the OR setting.

Another example, in the OR, is the positioning of the x-ray monitors or those used in endoscopic examinations, which may not be favorable to the surgeon’s positioning, increasing the need for constant adjustments during the procedure as well as an increased risk of injury for the patient caused by the associated muscular effort. It can also lead to loss of focus and prolongation of the surgical procedure [8] (also implying efficiency losses).

The concept of Extended Reality (XR) encompasses a spectrum of technologies, including virtual reality (VR), AR, and MR. Although these technologies may seem distinct, they share several common characteristics that unite them under the XR umbrella, namely digital interaction, digital visualization, immersion, and presence, with the goal of creating immersive and interactive digital experiences. Despite the recent prominence of XR, the term “mixed reality” was first introduced by Paul Milgram in 1994 to describe the merging of the physical and the digital worlds as part of a virtual continuum [9, 10].

Constant technological improvements in medicine, such as AR and MR, are redefining healthcare by blending digital and physical worlds to transform surgical practices, training, and patient outcomes. AR and MR are both forms of XR however they differ in the level of immersion and interaction they provide.

AR integrates computer-generated objects and virtual content into the real world, enhancing perception by overlaying digital elements onto physical environments and enhancing real-world images on flat screens without special devices [11]. For instance, AR Snapchat filters use face-tracking software and smartphone cameras to add puppy ears to a person’s face.

MR incorporates three critical features: combining real-world and virtual objects, real-time interaction, and mapping virtual objects onto physical ones to enable interactive experiences. MR achieves this integration of physical and digital realms through headsets and other devices, offering immersive 3D experiences [12]. MR not only overlays but also anchors virtual objects to the real world, enabling users to seamlessly interact with both environments. For instance, MR headsets can project holograms of people or objects that users can manipulate with gestures or voice commands. In other words, AR and MR are similar in that they both enhance the real world with digital content, but they differ in how they achieve it. AR adds 2D overlays to flat screens, while MR creates complex 3D interactions with immersive devices [13].

The use of MR in surgical contexts goes beyond being a mere innovation or trend. It represents an evolution in a continuous search for precision and effectiveness in the medical practice. MR offers an immersive and interactive platform that seamlessly combines the physical and digital worlds. This allows surgeons to navigate complex anatomical structures, practice procedures or plan surgeries with unparalleled detail and realism, while in a controlled environment.

The OR can also use MR as a surgical planning tool, a surgical approach assist, and as a teaching tool. This technology, for example, would enable learners to better prepare and flatten their learning curve through an immersive experience. It fills the void of information access and contact with other specialists that exists in the modern OR, making it easier for them to gain knowledge and expertise. The use of MR allows better accessibility, hands free, to all information, namely anatomical images, both during planning and in a surgical approach. In addition, when the surgeon is using a HMD, he is able to share with the team his personal view, which is paramount in minimally invasive procedures where usually only the main surgeon has a complete view of the operating field.

Several authors have recognized the innovative and beneficial approach of using technology, particularly MR, computer-aided assessment, and 3D models, in healthcare, particularly in medical and surgical training [14, 15]. These tools in the OR offer significant advantages, including real-time remote mentoring for trainee doctors during surgical procedures, immediate personalized feedback, expanded visualization of medical data in immersive environments for easier identification of anatomical anomalies, and detailed preoperative simulation. These immersive environments enhance training focus, improve anatomical understanding, and provide practical experience, transforming surgical practice and advancing medical education and healthcare quality. MR integrates patient-specific data with real-time observations, benefiting surgical training, education, and planning, particularly in minimally invasive surgery. Surgeons utilize MR to study human anatomy, plan surgeries, enhance procedure accuracy, and facilitate collaboration, thereby improving health outcomes and patient care [15, 16].

Mixed reality (MR) has been shown to improve surgical education by providing a higher quality educational experience, improving skill progression, and ensuring greater consistency in learning when compared to traditional teaching methodologies for basic surgical skills [17]. Additionally, a narrative review of the literature has highlighted the potential for MR to improve intraoperative accuracy, surgical outcomes, and patient satisfaction. According to, MR can enhance the assessment of surgical risks, enable modification of surgical strategy as necessary, and increase patient satisfaction when used during surgery [16]. Additionally, a systematic review concluded that augmented and mixed reality technology improve surgical outcomes by increasing navigational speed and reducing navigational errors during surgery [18]. Overall, MR technology has the potential to enhance surgical training, education, and planning, leading to improved surgical outcomes, accuracy of procedures, and patient care.

This study aims to review the predominant research on mixed reality applications in surgical settings from the last five years, to identify the trends, explore use cases, and evaluate both its applications and limitations.

### Research Question

Using the PICO framework (standing for Patient/Population/Problem, Intervention, Comparison, Outcome), a tool for formulating evidence-based research questions, a research question has been derived to better support the systematic review:

*"Among surgical teams (P), how does the incorporation of mixed reality tools (I) impact surgical procedures (O) when compared to conventional methods (C)?"*

This question will guide the search for content, organization of concepts and the extraction of information from the articles to be reviewed. In the Discussion section, the research question will be addressed and an answer, encompassing the main aspects of the topic will be provided.

### Document Structure

In this review article, the multifaceted world of MR in surgery, its diverse applications, its impact on surgeon training and education, its role in pre-operative planning, and its potential to enhance patient safety are the focus of exploration. Observing the referred problems, a systematic review on the use of MR in the OR was conducted to answer the research questions proposed.

## Methods

To elaborate this systematic review, the guidelines of the PRISMA statement (Preferred Reporting Items for Systematic Reviews and Meta-analyses) were followed [19]. From now on, the protocol of this study will be available on the PROSPERO platform and can be accessed with the following code: CRD42023427699.

This study aims to provide an up-to-date review of the evidence regarding the effectiveness of MR in the OR. To identify the research questions, the PICO strategy was used [20], Population: OR personnel; Intervention: the use of MR technology tools; Comparison: surgeries using MR tools versus conventional surgery and its traditional teaching and, Outcome: characterize the impact of the use of the tools on professional and patient experience while identifying the most relevant aspects (such as surgical duration, complication rate, ergonomics, learning, etc.)

### Search Strategy

**Fig. 1:**
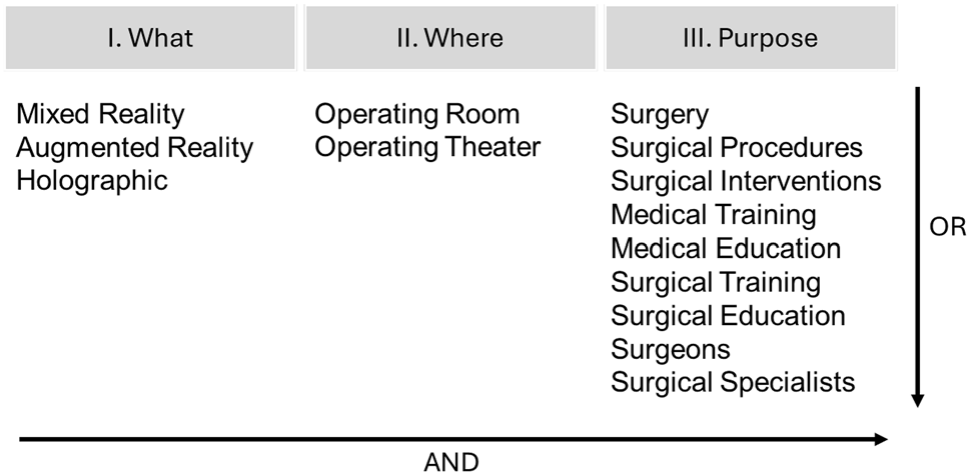
Keywords and logic strategy used in the development of the search query for article selection in the scientific repositories

To identify the relevant studies that allow to answer the research question, a search was conducted in the most reputed repositories using the search strategy depicted in Fig. 1.

The search query was organized as follows: ("Mixed Reality" OR "Augmented Reality" OR "Holographic") AND ("Operating Room" OR "Operating Theater") AND ("Surgery" OR "Surgical Procedures" OR "Surgical Interventions" OR "Medical Training" OR "Medical Education" OR "Surgical Training" OR "Surgical Education" OR "Surgeons" OR "Surgical Specialists"). The review primarily focuses on the use of mixed reality in the operating room. However, the search query also included the term “Augmented Reality” since its debatable definition is sometimes overlapped with mixed reality. The queried databases included PubMed, IEEE, ScienceDirect, ACM, Academic Search Complete, Web of Science, and Scopus. The article selection was based on the article’s title or abstract, and filtered by language (English and Portuguese only) and publication date (articles from 2018 beyond as of June 1^st^ 2023).

### Eligibility Criteria

The eligibility criteria for this systematic review were determined based on the population of the articles, their design, and characteristics. Articles must be written in either English or Portuguese, have the complete text available, and not be a review or conference article. They must have been published between 2018 and 2023 and address the use of MR in the OR (surgery planning is not considered part of the OR activity).

### Study Selection

**Fig. 2:**
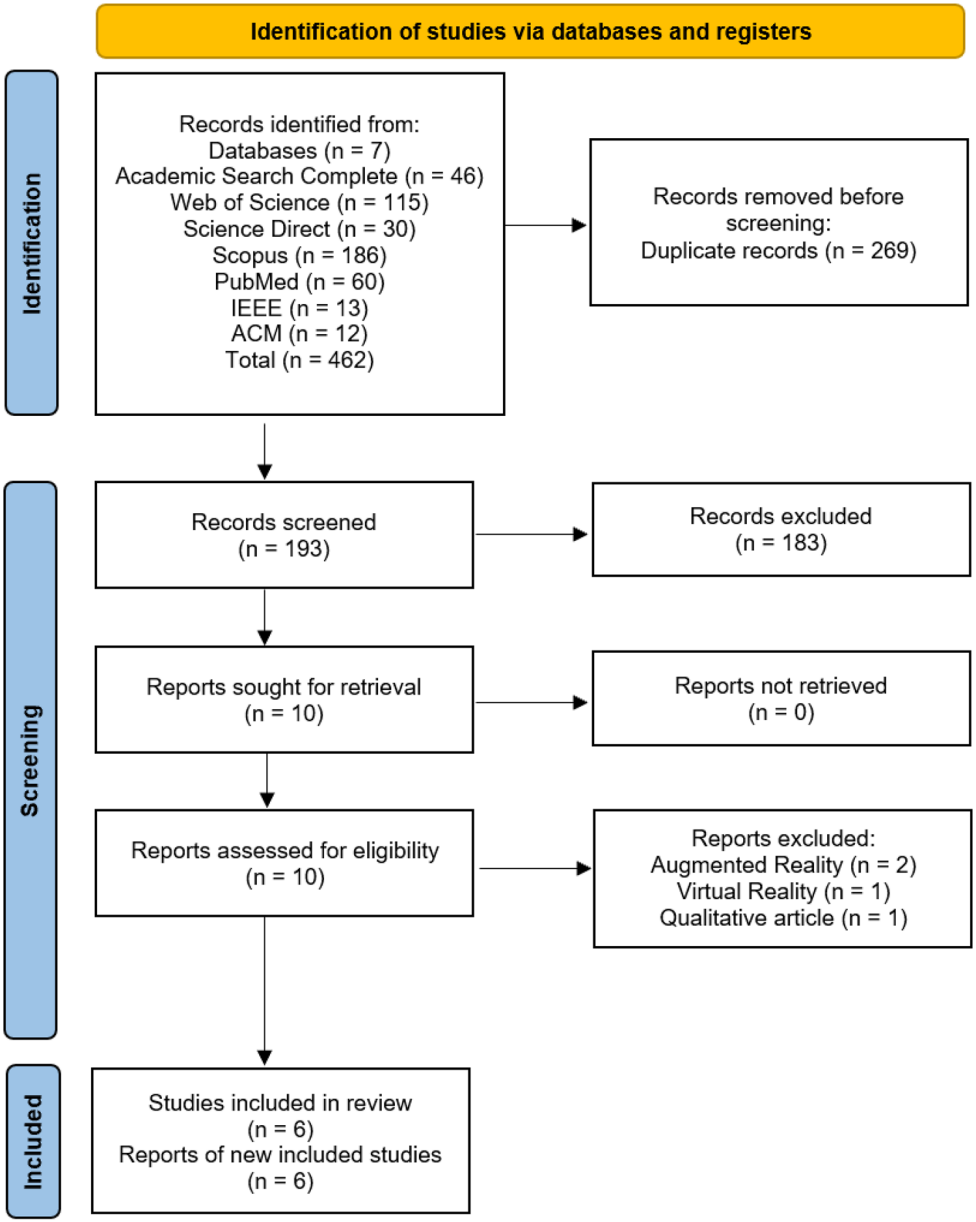
PRISMA flow diagram demonstrating included and excluded studies and the reasons for exclusion in the systematic review

The authors analyzed scientific articles in three stages, considering the title, abstract, and full text. They used inclusion and exclusion criteria and excluded duplicate articles. Next, two authors independently selected articles that met the review's objective and inclusion criteria by reading their titles and abstracts. The articles included by one reviewer, but excluded by the other, were later subject to analysis by a third party, who had the final decision on their inclusion. After analyzing each article using this method, a second review of the full text of the remaining articles was conducted. In cases where the two authors disagreed, the third was consulted again, until a final list was reached. This process is illustrated in Fig. 2, using the PRISMA diagram. Finally, the characteristics of each selected article were compiled in a table, which presents the conclusions on the subject.

### Quality Assessment in Systematic Reviews

The assessment of each article’s bias is made according to the type of study reported. Two main groups were set: one for articles referring to case series and another for case–control studies. The distinguishing factor between these two groups was the presence of a control group. The group of articles describing case series did not include a control group and measured values with comparison. In contrast, articles with a control group compared both groups. To assess the risk of bias in case series studies, the scale used was the one developed by the Canadian Institute of Health Economics (IHE) [21] was used, while the Newcastle–Ottawa Scale (NOS) [22] was chosen to assess the risk of bias in case control studies. The initial scale assesses the potential for bias by considering factors such as the study’s purpose, design, population, interventions, outcomes, statistical analysis, results, conclusions, and competing interests. The NOS employs a star rating system to evaluate the risk of bias, taking into account the study’s selection, comparability, and exposure. Each article underwent the appropriate assessment.

## Results

A total of 462 articles were collected from the electronic databases. Of those, 269 were duplicates, remaining 193 unique articles. The abstracts of these articles went through a review process, resulting in ten articles selected for full-text analysis. After analyzing the full text of these articles, four articles were excluded: two for describing VR instead of MR, one for utilizing AR instead of MR and one for being a qualitative article with no relevant measurements for further analysis.

### Study Characteristics

The use of MR applications in the OR can be divided into two types: those that aim to facilitate the learning process and those that are used to assist the surgeon during the procedure. Examples of the former include works [23] and [24], which demonstrate the potential of MR technology as a learning tool. The first work focuses on trainee mentoring, while the second covers the creation of a virtual environment to prepare and train individuals for the surgical setting. Cen et al. [25] describe the use of MR for the 3D visualization of the patient’s organs in a perioperative context, providing support for the surgeon.

However, there are many challenges with MR. As a new technology, users are hesitant and need time to learn how to use it [24], which requires training and adaptation to new approaches. In addition, the preparation of MR content and programs is more difficult than for other established technologies [23], again requiring a step-up program for participants to gain expertise. The use of head-mounted displays also causes dizziness in some users [25]. This technology also has advantages, such as a lower cost of implementation when compared to other teaching tools in the OR [23], with satisfactory ratings from trainees, but also a new way to evaluate and prepare for different scenarios that occur in the OR [24]. In order to support the surgeon and help the surgeon understand and visualize some complex cases, MR is used to reveal 3D models of organs and their surrounding structures, facilitating the approach [25].

In the retrieved articles, five of the six were case series, which means that there was no control group with which to make a comparison. However, in these cases, measurements were made to evaluate the work performed, such as Likert scales regarding experience, established scales to evaluate performance, and measurements of patients whose surgical procedure used this type of technology.

The main characteristics of the six articles reviewed above are presented in Table 1. Still regarding the reviewed articles, in terms of the technology used to support MR, the Head-Mounted Displays (HMD) or Optical See-Through Head-Mounted Displays (OST-HMD) dominate the field, with Microsoft HoloLens® and the Magic Leap® being the most used. In Table 2, it is possible to compare the reviewed solutions in terms of the quality of their results, without forgetting the provided performance metrics. Finally, in Table 3, a cost–benefit analysis is presented for each case, considering the related challenges and limitations.

**Table 1:** Summary of the reviewed articles showing the application and related supporting technology

**Study**	**Country**	**Technology**	**Areas of application**
**Simone et al. **[23]	Italy	HMD	Remote mentoring
**Stefan **[24]	Germany and Austria	HMD	Competency assessment of professionals
**Cen **[25]	China	HoloLens	Assisting tool to cardiac surgery
**Saito **[26]	Japan	HoloLens, HoloeyesXR and Magic Leap 1	Intraoperative support system: 3D holographic cholangiography in hepatobiliary surgery
**Cartucho and Shapira **[27]	UK/Switzerland	HoloLens	Image-guided surgery
**Galati et al. **[28]	Italy	HoloLens	Open abdomen surgery

**Table 2:** Summary of the reviewed articles showing the proposed solutions, their related results and the considered performance metrics

**Study**	**Metrics**	**Results**	**Implemented solutions**
**Simone et al. **[23]	Likert-type scale questioning the students about the experience	Well accepted by the trainees	Mentoring students remotely using Mixed Reality
**Stefan **[24]	OSATS and OTAS assessment scores	Established method to evaluate the intraoperative performance	Use of a mixed-reality environment to simulate the surgical procedure
**Cen **[25]	Surgery duration, patient’s stay duration and recovery, RV to aortic peak systolic pressure ratio and change in baseline oxygen saturation	Easier to understand the surgical procedure and more interactive and simpler for trainees	Perioperative assistive tool during surgery, for visualization
**Saito **[26]	Qualitative analysis	Operators can move the hologram from the respective operators’ angles by means of easy gesture-handling without any monitors, and several surgeons wearing HMDs can share the same hologram. A more accurate reappearance of the bile duct can decrease the surgeon’s stress level and facilitate the performance of a safer and more precise operation	3D holographic cholangiography; remote medical education sessions
**Cartucho and Shapira **[27]	Usability questionnaire filled out by surgeons and subsequently analyzed	Improve surgical outcomes by providing real-time guidance and enhancing the surgeon's understanding of the patient's anatomy	MR visualization platform which projects multiple imaging modalities to assist intraoperative surgical guidance
**Galati et al. **[28]	Table with user feedback on the procedure with and without HoloLens	It can increase the execution speed by allowing multitasking procedures, by checking medical images at high resolution without leaving the operating table and the patient	Visualize information about the results of medical screenings, such as radiography, blood tests, and magnetic resonance imaging; visual information on the patient’s body by using mixed reality tools; sharing information with other professionals, this being useful for training, remote tutoring, and for receiving external advice from other physicians

**Table 3:** Summary of the reviewed articles showing the obtained effects, the inherent cost, the related challenges, and their respective limitations

**Study**	**Effects and costs**	**Challenges and limitations**
**Simone et al. **[23]	Low-cost implementation that allows remote teaching	Complex technical tuning
**Stefan **[24]	Possibility to evaluate intraoperative competences, in an immersive simulation	Lack of confidence with the technology, hesitation while during observation
**Cen **[25]	Facilitates the surgical planning and more dynamic than 3D printed models	Harder to learn for older professors and dizziness. Imaging techniques require contrast
**Saito **[26]	Better accuracy, the operator could perform the dissection more safely with better imaging; improved observation of the 3D biliary anatomy from various angles and sharing of the same hologram from the respective operators' angles; it revealed several new intraoperative findings regarding the biliary anatomy	3D holographic cholangiography; remote medical education sessions
**Cartucho and Shapira **[27]	Scrolling through volumetric data and adjusting the virtual objects transparency to avoid obstructing the surgeons view of the operating site	MR visualization platform which projects multiple imaging modalities to assist intraoperative surgical guidance
**Galati et al. **[28]	It can increase the execution speed of surgical procedures by allowing multitasking procedures, such as checking medical images at high resolution without leaving the operating table and the patient	Visualize information about the results of medical screenings, sharing information with other professionals, this being useful for training, remote tutoring, and for receiving external advice from other physicians

### Keyword Identification and Frequency

**Fig. 3:**
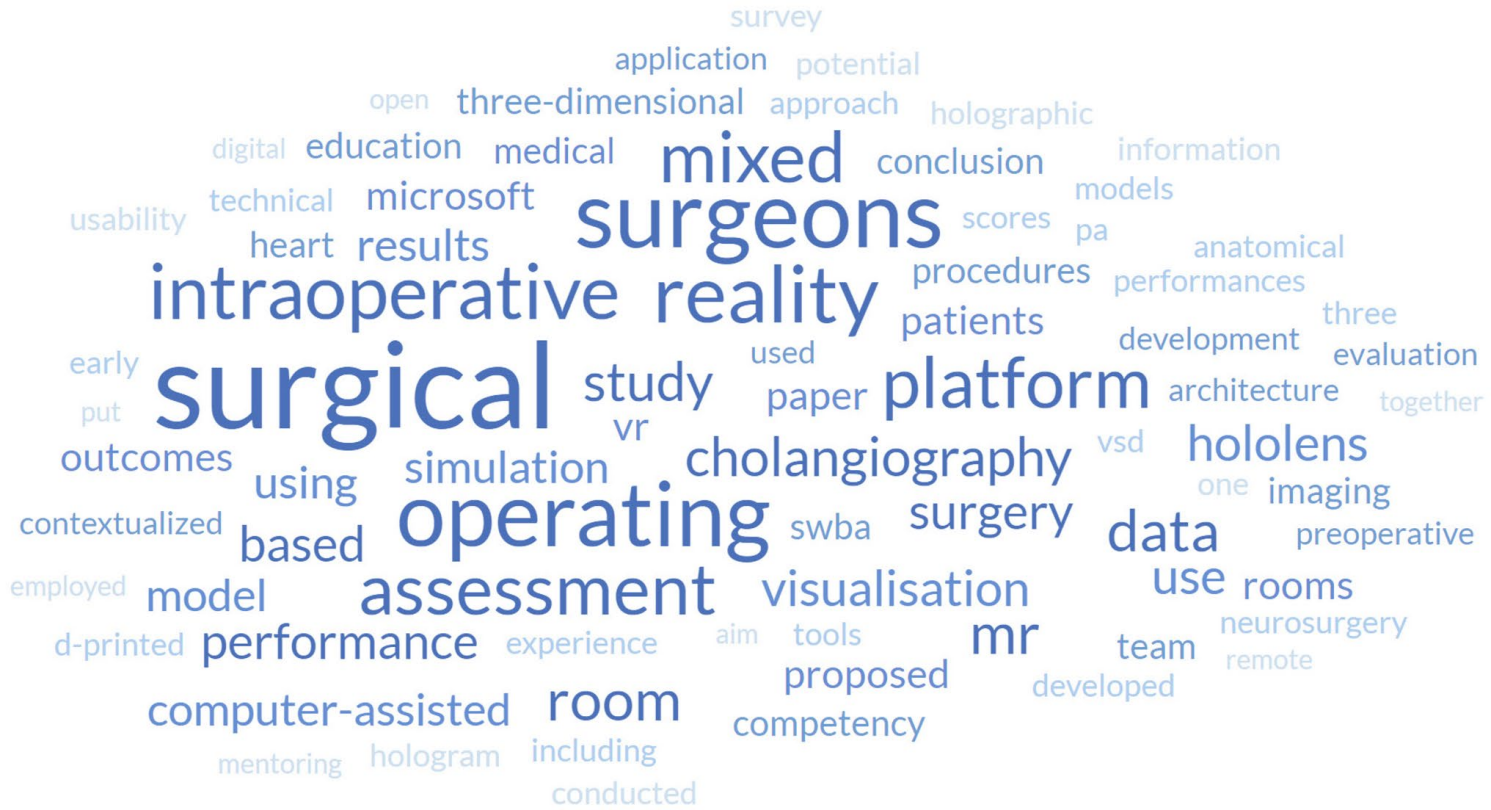
Word cloud based on the reviewed articles related keywords

Based on the abstracts of the reviewed articles, the most common words were selected and a word cloud was created by counting the frequency of each word, as show in Fig. 3. Word clouds are powerful tools that allow observing aspects such as key themes, trends, conceptual relationships, or methodological focus, among others. Looking at Fig. 3, as expected, words related to the application of the current research area are clearly visible (“operating”, “surgical”, “surgeons”), as well as those related to the associated technological concepts (“mixed”, “reality”, “platform”). It is interesting to note that words related to the benefits often associated with MR are well represented (“performance”, “assessment”, “visualization”, “study”). The term “intraoperative” appears frequently, underscoring the benefits of MR technology in surgical settings, validating focus of the approach and pointing to the relevance of the selected articles to the topic under study. Terminology related to the underlying operating mechanisms are also present (“computerized”, “simulation”, “data”), highlighting their importance as crucial components. Finally, from a technological perspective it is also possible to observe that Microsoft is the sole software company listed and that, besides Hololens, no other HMD is covered.

### Quality Assessment

**Fig. 4:**
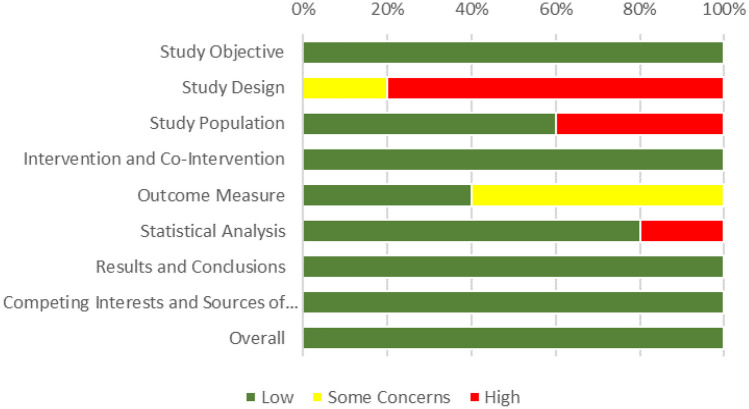
Percentage of risk of bias for the reviewed articles (according with the Canadian Institute of Health Economics case series approach [16])

The risk of bias of reviewed articles, summarized in Table 4, was assessed according to their respective study organization and the information available in the document, using two different scales. In the first scale, related to the case series articles, all the selected articles were declared to have an insignificant risk of bias. However, some of the aspects assessed did not have such an insignificant risk. For example, for study design, all articles had a considerable risk of bias. The selection of studies was not as unbiased as desired, as it were often carried in the same institution and not continuously. Although this may seem alarming, the reason for this is the low availability of MR equipment, resulting in few cases of use to study. Some articles did not have a low risk of bias in the study population, mainly because of poor description of the selection methods [23, 27]. The measures used to assess the outcome were not very well designed, resulting in a moderate risk of bias in the majority of the selected articles. One of the main problems was that the assessment of success was not done blindly, since the assessor was aware of the intervention used [24–27]. In some cases, the measurements made after the intervention and not before [24–27]. One of the reviewed articles also has a considerable risk of bias in the statistical analysis, as there were no appropriate relevant outcomes assessed with statistical tests. For parameters that were not reported, the articles presented a low risk of bias, which is why the risk of bias was assessed as low for all of them, as can be seen in Fig. 4.

**Table 4:**
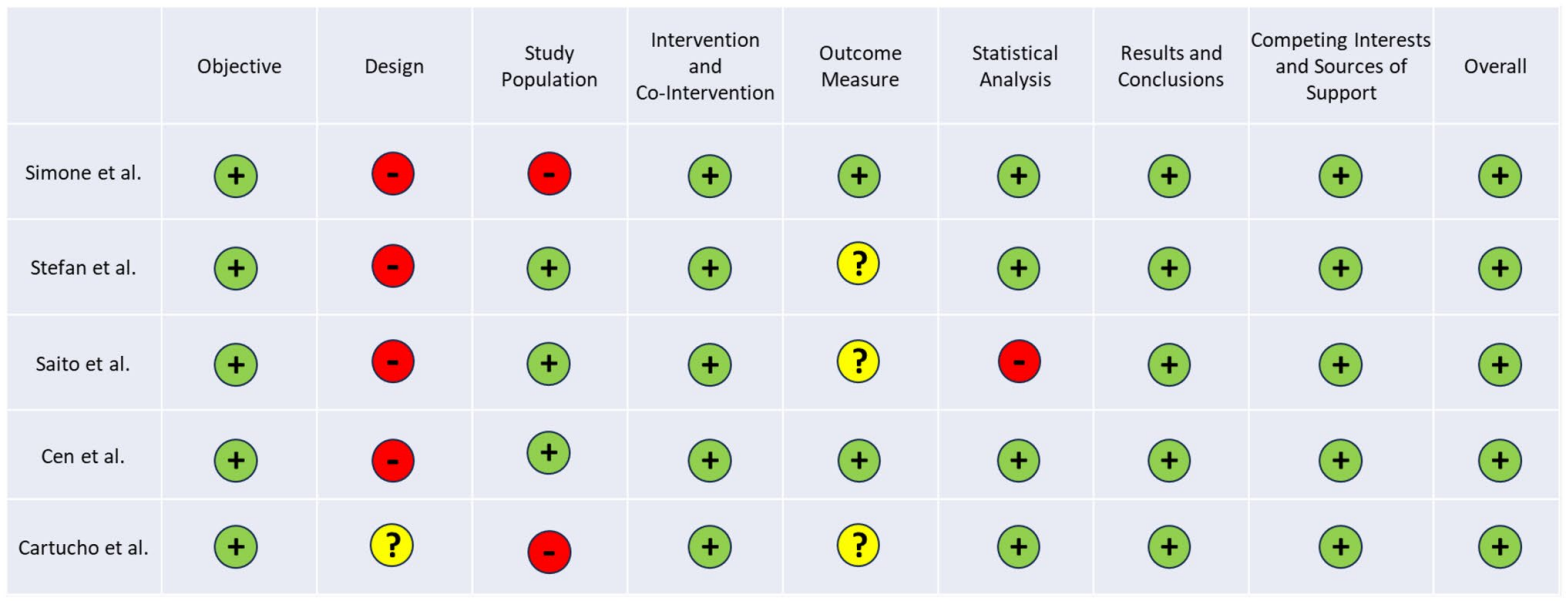
Bias assessment for the reviewed articles (based on Canadian Institute of Health Economics case series approach [21])

The system used for the remaining case–control study was the NOS [22]. Details of the star template are presented in Table 5. We found that the case definition included references to primary record sources in the selection criteria, which explains the missing star. Comparability and exposure had the maximum number of stars, which shows a good comparability of cases and controls based on the analysis and structured interview, both of which went blind to case/control status. Eight stars (out of a total of 9) were attributed to the case–control study.

**Table 5:** Quality assessment of the case control study article (according to the Newcastle–Ottawa Scale [22])

**Study**	**Selection (4)**	**Comparability (2)**	**Exposure (3)**	**Total (9)**
**Galati et al.** [28]	☆ ☆ ☆	☆ ☆	☆ ☆ ☆	8

The results indicated that most of the data in this systematic review came from studies of high quality.

## Discussion

### Highlights

The articles reviewed highlight various benefits and challenges associated with the implementation of innovative technologies in a surgical setting. According to Simone et al. [23], MR proved to be a valuable tool for continuing education, allowing remote mentoring, enhanced visualization of medical data, while providing a better identification of anatomy and related anomalies. Stefan [24], in their study, explores simulated workplaces and discusses a comprehensive approach to computer-assisted assessment, emphasizing empirical validity, realistic contextualization, and the ability to provide immediate feedback. Cen [25] demonstrates the wide application range of MR in OR by the use of 3D cardiac models in conjunction with VR and MR to provide better anatomical understanding, surgical simulation, and intraoperative decision support. In addition, Saito [26] emphasizes standardization and contextualization in surgical performance assessment, supported by evidence of validity in different domains. Cartucho and Shapira [27] proposes an integrated platform for multiple imaging modalities, with an interactive 3D model and innovative functionalities, particularly applicable to vascular neurosurgery. Galati et al. [28] highlights the utility of MR for training and telementoring, emphasizing improved efficiency and image quality.

Based on the reviewed articles, MR technology offers significant advantages in the operating room, enhancing the overall surgical experience and outcomes. MR allows surgeons to visualize anatomical structures in 3D and superimposed images on the actual patient, which facilitates the understanding of complex anatomy [25–28]. This capability not only aids in precise preoperative planning, by allowing detailed preparation and simulation, but also enhances surgical accuracy with real-time overlays during procedures [25–28]. Additionally, MR can reduce operative time by providing precise, real-time information, thus improving safety and efficiency [25–28], which are highly relevant for the patient, for the surgical team and ultimately for the healthcare institution. This technology is also invaluable for medical training, not only offering realistic simulations while enabling instant feedback, but also allowing real-time remote collaboration and support for continuous learning [23–25]. These features collectively contribute to an effective reduction in medical errors and support to an integrated approach to healthcare, seamlessly connecting with other electronic health systems [23–28].

Table 6 summarizes the main advantages and highlights the multifaceted benefits of utilizing mixed reality (MR) in medical education and practice.

**Table 6:** Summary of the identified advantages

**Advantage**	**… from Discussion**
Training and remote mentoring	MR proved to be a valuable tool for continuing education, allowing remote mentoring, enhanced visualization of medical data, and identification of anatomical anomalies [23]. The utility of MR for training and telementoring, emphasizing improved efficiency and image quality [28].
Contextualization	A comprehensive approach to computer-assisted assessment, emphasizing empirical validity, realistic contextualization, and the ability to provide immediate feedback [24].
3D Models	the use of 3D cardiac models in conjunction with VR and MR to provide better anatomical understanding, surgical simulation, and intraoperative decision support [25].
Integration with Platforms	an integrated platform for multiple imaging modalities, with an interactive 3D model and innovative functionalities, particularly applicable to vascular neurosurgery [27].
Reduced Learning Curve	simulation shortens the learning curve and is more cost-effective and time-efficient than traditional methods. Simulation in a safe environment also shown to be crucial for skill development and retention [23–25].
Access to Critical Data	These glasses can provide precise, real-time gesture guidance to help the surgeon navigate and perform procedures with greater accuracy. The ability to access critical clinical process data is another benefit, displaying important information such as medical images, diagrams or patient data without the need to divert attention to monitors or external devices [25, 26].
Real-Time Communication	Real-time communication could be facilitated, allowing instant collaboration with other healthcare professionals during the procedure [23, 28].

In the process of conducting this systematic review, several limitations were identified in the use of MR in the OR. These limitations, detailed in Table 7, span various dimensions, including cost, time, technology, adherence to guidelines, privacy and ethics, among others, can constitute challenges for the introduction and operation of this new technology.

**Table 7:** Summary of the identified limitations

**Limitation**	**… from Discussion**
High Cost	The cost aspect of Mixed Reality (MR) solutions is examined, with special reference to a low-cost implementation…indicating a wide range of associated costs [23].
Time and Training Adaptation	Healthcare professionals need time and training to adapt to the use of these new technologies, which can be challenging [23–25].
Technological Dependence	There can be excessive dependence on technology, which can be problematic if technical failures occur during surgery [25–28].
Interference with Procedures	Mixed reality equipment can be bulky or interfere with the physical space of the operating room [23–25].
Data Integrity Issues	Incorrect or outdated data can lead to serious errors during surgery [25–28].
Privacy and Security	The use of connected technologies can raise privacy and security concerns regarding patient data [25–28].
Image Reliability	The accuracy of the generated images can be compromised by factors such as patient movement or sensor failures [25–28].
Fatigue and Discomfort	Prolonged use of mixed reality devices can cause eye fatigue and discomfort for healthcare professionals [23–25].

However, the implementation of MR in the operating room has yet some open challenges. The cost of MR systems, including hardware and software, can be a significant barrier for many healthcare institutions [23]. Additionally, healthcare professionals may require substantial training and time to adapt to these new technologies, which can be a daunting task [23–25]. The reliance on technology also poses risks, such as an eventual technical failure during critical procedures [25–28]. Furthermore, issues such as data integrity, privacy, and security are paramount concerns, as MR systems often involve the handling of sensitive patient information [25–28]. Physical interference with surgical procedures and the potential discomfort caused by prolonged use of MR devices are additional identified limitations [23–25]. Lastly, the fast evolution of technology may require frequent updates, adding to the long-term costs and complexity of maintaining MR systems in the surgical environment [23].

The summary presented in Table 7 provides a comprehensive overview of these limitations, offering a clear framework for understanding the encountered challenges.

MR technology offers a broad range of application possibilities. In the context of OR and based on the reviewed articles, a summary of the main areas where MR can have an impact is presented in Table 8.

**Table 8:** Studies coverage grouped by contextualization area

**Contextualization Area**	**Studies**	**MR Applications**
Education, Training, Simulation and remote mentoring	[23, 24, 28]	Education, Training and Simulation. Remote mentoring.
Visualization and Modeling	[23, 25, 27]	Enhanced visualization. Interactive 3D models. Anatomical understanding.
Intraoperative Guidance and Support	[23, 25, 27]	Real-time guidance. Intraoperative decision support.
Assessment and Feedback	[24, 26]	Computer-assisted and performance assessment. Immediate feedback.
Specialized Applications	[25, 27]	Specific surgical applications.
Technological Integration	[27]	Integration of multiple imaging modalities.

In summary, mixed reality technology can provide several benefits during a surgical procedure. Some of these advantages include augmented visualization, which allows direct visual information to be superimposed on the surgeon’s field of view, providing an improved perspective of anatomy and surgical instruments.

In addition, HMD can provide precise, real-time gesture guidance to help the surgeon navigate and perform procedures with greater accuracy. The ability to access critical clinical process data is another benefit, displaying important information such as medical images, diagrams or patient data without the need to divert attention to monitors or external devices. Real-time communication could be facilitated, allowing instant collaboration with other healthcare professionals during the procedure, either participating in the same room or participating remotely.

Finally, HMD and MR technology can be useful in education and training, allowing medical trainees and students to observe procedures in real time, from the unique surgeon’s perspective, and gain practical experience.

### Answer to Research Question

In the introduction of this manuscript a research question was defined, as transcribed here "Among surgical teams (P), how does the incorporation of mixed reality tools (I) impact surgical procedures (O) when compared to conventional methods (C)?".

A beneficial impact is mentioned by several authors. In fact, from the four studies that addressed the use of MR during surgery [25–28], in all cases, there is evidence of benefits compared to conventional surgery. These benefits encompass easier understanding of procedures, increased accuracy, improved safety, and reduced operative time, among others.

The cost effectiveness of a specific procedure also impacts the surgery, which is mentioned in most articles. In [23], the cost aspect of Mixed Reality (MR) solutions is examined, with special reference to a low-cost implementation. The other studies within the selected literature explore different supporting architectures, each serving different purposes and indicating a wide range of associated costs. It is noteworthy, however, that the dynamic nature of the technology evolution and the expected decrease in the cost of hardware devices suggest a trend toward increased performance. This trajectory points to a prospective reduction in the barriers to access MR solutions, highlighting the potential for increased affordability and widespread availability in the future. From an institution’s perspective, given these benefits, investing in MR solutions may prove to be the most appropriate way to reduce surgery time while improving surgical outcomes, contributing to reducing overall operational costs.

Another relevant impacting factor, covered by three studies [23–25] is the ability to use MR technology for learning. The authors of these studies agree that simulation shortens the learning curve and is more cost-effective and time-efficient than traditional methods. Simulation in a safe environment also showed to be crucial for skill development and retention. The ability to teach remotely and share perspectives is also a highly valued benefit of MR. In addition, MR provides assessment methods for immediate feedback and allows for scenario repetition to track performance improvement [29].

### Opportunities for MR use in OR

The integration of mixed reality (MR) technologies in operating rooms presents substantial promise for enhancing surgical precision, training, and overall patient outcomes. However, one of the critical barriers to the widespread adoption and effective utilization of these technologies is the challenge of interoperability between different systems. Mixed reality systems often rely on a complex amalgamation of software and hardware components, including headsets, sensors, imaging devices, and surgical instruments. These components are frequently developed by different manufacturers, each with their own proprietary protocols and standards. This lack of standardized communication protocols hinders seamless integration and data exchange between devices, leading to inefficiencies and potential errors during surgical procedures.

For instance, current MR systems might struggle to synchronize real-time data from disparate sources such as patient monitoring systems, radiologic imaging, and surgical navigation tools. This discordance can result in delays or inaccuracies in the information presented to the surgeon, thereby affecting decision-making processes. Furthermore, the need for manual data input or adjustments due to non-compatible systems diverts the surgeon's focus from the patient, potentially compromising the quality of care. Addressing interoperability issues requires the development and adoption of universal standards and protocols that facilitate seamless data exchange and integration across various MR systems and devices.

Additionally, current MR hardware presents limitations that constrain its practical application in the operating room. One notable limitation is the bulkiness and weight of existing MR headsets. These devices can be cumbersome, leading to discomfort during prolonged use and potentially impeding the surgeon's dexterity and range of motion. Future MR hardware needs to be lightweight, ergonomically designed, and adaptable to long surgical procedures without causing strain or fatigue.

Another critical area for improvement is the precision of finger tracking and hand gestures. Accurate finger tracking is essential for surgeons to manipulate virtual objects, navigate through medical images, and interact with MR interfaces effectively. Current systems often suffer from latency issues and lack the fine motor control required for delicate surgical tasks. Advancements in sensor technology and machine learning algorithms could enhance the accuracy and responsiveness of finger tracking, making MR interfaces more intuitive and reliable for surgical use.

Moreover, the practicality of MR hardware in sterile environments remains a challenge. Devices must be easily sterilizable or designed to maintain sterility throughout procedures. Future MR solutions could incorporate materials and designs that facilitate quick and effective sterilization, ensuring compliance with stringent surgical hygiene standards.

The integration of haptic feedback in MR systems is also important, since it could significantly enhance their utility in the operating room. Haptic feedback provides tactile sensations that can simulate the feel of tissues and instruments, offering surgeons a more immersive and informative experience. While current MR hardware typically lacks this capability, future developments could incorporate advanced haptic technologies to replicate the tactile feedback necessary for intricate surgical procedures.

### Study Limitations

Despite the rigorous methodology that was employed in this systematic review, several limitations must be acknowledged:

1. Search Strategy Limitations: Our search was confined to six major databases and did not include a comprehensive search of grey literature, which may have led to the exclusion of relevant studies not indexed in these databases. Furthermore, we restricted our search to English-language publications, potentially introducing language bias. In addition, the results are obtained from an initial search query that involves a personal selection of keywords. Distinct search terms can lead to distinct results.
2. Publication Bias: There is an inherent risk of publication bias, as studies with positive findings are more likely to be published than those with null or negative results. This bias could have influenced our overall findings.
3. Study Selection and Inclusion Criteria: The inclusion criteria were designed to ensure relevance and quality, yet they may have excluded pertinent studies, especially those with broader or slightly differing focuses. Additionally, the included studies exhibited considerable heterogeneity in terms of methodologies and outcome measures, which complicates direct comparisons and synthesis of results.
4. Quality of Included Studies: The quality of the included studies varied, with some studies exhibiting distinct methodological approaches. This variability can impact the confidence in the pooled results.
5. Data Extraction and Synthesis: Although a rigorous data extraction process has been employed, there remains the possibility of human error. The diversity in study designs and outcome measures presented challenges in synthesizing the data, necessitating cautious interpretation of the pooled findings.
6. Generalizability of Findings: The generalizability of our findings is limited by the specific populations and settings of the included studies. As most studies were conducted in high-income countries, the applicability of the results to low- and middle-income settings is uncertain since the cost of HMD and MR technology can still be a limiting factor.
7. Temporal Limitation: Our search strategy included studies published between 2018 and 2023. Given the rapidly evolving nature of this field, it is possible that newer studies have emerged that were not included in our review.
8. Number of articles: While a revision based on a modest selection of six articles may offer a limited scope, it remains a valuable endeavor, especially given the timeliness of the topic under consideration. Collectively, the six selected articles help provide an initial overview that helps to describe the current landscape of the topic. This focused examination allows for a nuanced exploration of the benefits, limitations, and emerging opportunities associated with the topic.

Also, the inherent novelty of the topic, coupled with the insights provided by these selected articles, enhances the current topic understanding and lays the groundwork for further comprehensive research. As the field evolves, these initial findings can serve as a foundational framework for future research, guiding scholars toward a deeper understanding of the intricacies surrounding the topic.

By transparently acknowledging these limitations, the authors aim to provide a balanced context for the interpretation of the reported findings and guide future research efforts in this domain.

## Conclusions

### Mixed Reality in the Operating Room

Along this review, the aim was to answer the proposed research question and determine the advantages and disadvantages of incorporating MR tools in the OR. After analyzing the articles, the use of MR can be considered a helpful tool in many areas of the OR, ranging from the using the device to visualize intraoperatively, to communicating with the exterior for assistance. Many of the reviewed papers, describe the MR as a way to extend the field of view of the surgeon, adding a screen containing what is seen in the monitors regularly placed in the OR, from endoscopic imaging, but also other information of the patient, such as radiographies, blood tests, and other types of medical imaging [25–28]. Another common application of MR in the OR, as seen in the reviewed articles, is its use as a communication tool that can be used for mentoring, training and clarification of concepts from other specialists [23, 26, 30].

When using this technology, the users faced many challenges such as adapting to a new technology, which takes time [26], especially for older users [25], lack of confidence in this type of new technology [24]. Some of the researchers faced difficulties in developing software for MR, judging the tuning as slow [23], others argued the low capability of tracking from the device [26], its parallax error and the headset autonomy [24]. In the area of ergonomics, some articles described the use of MR devices as inducing headaches or dizziness [25], as well as reduced field of view and parallax error [28].

The use of this technology is considered as a cheaper alternative to the existing methods but also a tool of great quality, being, overall, well accepted by the questioned trainees [23]. This is seen as a way to prepare the residents for real scenarios, digitally, complementing what is the standard today, providing better training and improving education on surgical tactics and methods [24, 31]. It is well known that simulation can be a powerful tool to support learning, especially in the healthcare area [29]. In the surgical procedure field, the visualization of 3D models related to the surgery are presented as a benefit for a better understanding of the condition and situation by the novice surgeons, offering different degrees of immersive experience [25]. MR technology also allows for a training environment appropriate for recreating realistic and reproducible scenarios without putting the patient at risk [24]. The use of MR contributed to improved imaging which can decrease the surgeon’s stress level and facilitate the performance of a safer and more precise operation [22]. Compared to the visualization options currently available, it enables the surgeon to consult, control, manipulate data for better decision making and remain sterile [23]. Another useful aspect is the possibility of combining MR functions such as video recording for training and displaying image, text, video, or 3D objects as guidance [24]. The capability to load several DICOM data into the surgeon’s field of view in real time is also a particularly useful feature [24]. Reducing the problems with intraoperative visibility, MR applications tend to lower the procedure time, allowing multitasking and improving real-time guidance, within this context [26–28].

While still evolving, these immersive technologies have the power to bridge the gap between aspiration and reality, turning the dream of an improved, patient-centered surgical experience into a tangible reality.

### Open Challenges and Future Directions

While MR technology holds enormous potential, its current state of development presents challenges, particularly in the unique context of the operating room. Achieving regulatory approval for use in the OR is critical to widespread adoption, ensuring compliance with rigorous safety and efficacy standards. Practical considerations related to supporting hardware, such as limited field of view, battery constraints during long surgeries, and the need for extended user comfort, even during periods of hardware inactivity, are critical areas for ongoing development.

In addition, the need for fast processing hardware to ensure low latency and reliable synchronization for efficient data acquisition and communication with external devices is crucial. Overcoming these technical issues is essential to minimize adverse effects such as dizziness or motion sickness and to enhance the realistic and precise visualization and manipulation of virtual objects, despite potential limitations.

Finally, promoting software interoperability between Head-Mounted Displays (HMDs) and medical devices could significantly expand the applications of MR in the OR.

These challenges will soon be overcome as MR technology becomes a dependable and essential tool in the surgical setting.

The use of such technologies will be integrated with other evolving fields, such as Video Assisted Thoracic Surgery (VATS) and Robotic Assisted Thoracic Surgery (RATS), due to its ability to provide critical information and guidance during procedures.

The rise of artificial intelligence and its growing “understanding” of the real world may also open the possibility for MR technology to play a more active role in surgery.

## Data Availability

No datasets were generated or analysed during the current study.
